# Prevalence of sexually transmitted infections (STIs), associations with sociodemographic and behavioural factors, and assessment of the syndromic management of vaginal discharge in women with urogenital complaints in Mozambique

**DOI:** 10.3389/frph.2024.1323926

**Published:** 2024-04-18

**Authors:** Alice Manjate, Gladys Sergon, Darlenne Kenga, Daniel Golparian, Yuriy Tyulenev, Osvaldo Loquilha, Fabião Mausse, Alexander Guschin, José Carlos Langa, Alfeu Passanduca, Jahit Sacarlal, Magnus Unemo

**Affiliations:** ^1^ School of Medical Sciences, Faculty of Medicine and Health, Örebro University, Örebro, Sweden; ^2^ Departament de Microbiologia, Faculdade de Medicina, Universidade Eduardo Mondlane, Maputo, Mozambique; ^3^ WHO Collaborating Centre for Gonorrhoea and Other Sexually Transmitted Infections, Faculty of Medicine and Health, Örebro University, Örebro, Sweden; ^4^ Department of Healthcare, Moscow Scientific and Practical Center of Dermatovenereology and Cosmetology, Moscow, Russia; ^5^ Departamento de Matemática e Informática, Faculdade de Ciências, Universidade Eduardo Mondlane, Maputo, Mozambique; ^6^ Institute for Global Health, University College London (UCL), London, United Kingdom

**Keywords:** prevalence, sexually transmitted infections, syndromic management, vaginal discharge, Mozambique

## Abstract

In Mozambique, sexually transmitted infections (STIs) are estimated to be prevalent, but diagnosis and treatment of curable STIs rely only on syndromic management. We examined the prevalence of four non-viral STIs and HIV-1/2, based on etiological diagnosis, associations with sociodemographic and behavioural factors, and the STI diagnostic accuracy of the vaginal discharge syndromic management in women with urogenital complaints in Maputo, Mozambique. A cross-sectional study was performed in Maputo, Mozambique, February 2018–January 2019, enrolling 924 women of reproductive age with urogenital complaints. Endocervical/vaginal swabs were sampled and chlamydia, gonorrhoea, trichomoniasis and *Mycoplasma genitalium* infections were diagnosed using a multiplex real-time PCR (AmpliSens; InterLabServices). Serological testing was performed for HIV-1/2. A structured questionnaire collected metadata. All data were analyzed in STATA/IC 12.1 using descriptive statistics, chi-square tests and logistic regression model. About 40% of the women were less than 24 years old, 50.8% were single, 62.1% had their sexual debut between 12 and 17 years of age, and the main complaint was vaginal discharge syndrome (85%). The prevalence of chlamydia was 15.5%, trichomoniasis 12.1%, gonorrhoea 4.0%, *M. genitalium* 2.1%, and HIV-1/2 22.3%. The vaginal discharge syndrome flowchart had a sensitivity of 73.0%–82.5% and a specificity of 14%–15% for the detection of any individual non-viral STI in women with urogenital complaints. In total, 19.2% of the symptomatic women with chlamydia, trichomoniasis or gonorrhoea would not be detected and accordingly treated using the vaginal discharge syndromic management (missed treatment) and 70.0% of the women would be treated despite not being infected with any of these three STIs (overtreatment). In conclusion, a high prevalence of especially chlamydia, trichomoniasis, and HIV-1/2 was found in women of childbearing age with urogenital complaints in Maputo, Mozambique. Syndromic management of vaginal discharge revealed low accuracy in the detection of STIs in symptomatic women, especially low specificity, which resulted in under-treatment of STI-positive cases and incorrect or over-treatment of women with urogenital complaints, many of whom were negative for all the non-viral STIs. Etiological diagnosis is imperative for effective management of STIs in symptomatic and asymptomatic women.

## Introduction

Sexually transmitted infections (STIs), including HIV-1/2, remain neglected public health problems with a significant burden of mortality and especially morbidity worldwide (1–4). The World Health Organization (WHO) estimates that more than 1 million new global cases of curable STIs are acquired every day, i.e., 374 million estimated global cases of trichomoniasis (etiological agent: *Trichomonas vaginalis*), chlamydia (*Chlamydia trachomatis*), gonorrhoea (*Neisseria gonorrhoeae*), and syphilis (*Treponema pallidum* subspecies pallidum) among adults in 2022 (3, 4). Sub-Saharan Africa is the most affected subregion, with about 40% of the global burden of non-viral STIs, i.e., approximately 60 million new gonorrhoea, chlamydia and trichomoniasis cases estimated annually, with the 15–24 years age group being the most affected (5–7). In Mozambique, the estimated prevalence of non-viral STIs, based on interviews in the population of reproductive age, was in 2015 7% among women and 5% among men (8), however, no STI prevalence or incidence data based on etiological diagnosis of STIs are available. Furthermore, Mozambique is among the top ten countries in the world with the highest HIV-1/2 prevalence rates (9).

Undetected, untreated, and/or inappropriately treated STIs can lead to complications and sequelae, which disproportionally affect women, including pelvic inflammatory disease, ectopic pregnancy, foetal or neonatal death, premature birth, infertility, and increased acquisition and transmission of HIV-1/2 (1, 7, 10). Early diagnosis and appropriate treatment are essential for the prevention of transmission, complications, and sequelae of STIs. However, in countries with limited resources, etiological diagnosis of most non-viral STIs (syphilis is the main exception) remains difficult due to the lack of facilities, qualified technical personnel and financial resources (11, 12).

In 1985, due to the limited access to and funding for etiological STI diagnosis in many less-resourced settings, WHO published the first guidelines regarding syndromic management of four common STI syndromes (urethral discharge, vaginal discharge, genital ulcers, and lower abdominal pain or pelvic inflammatory disease). Current WHO guidelines address six syndromes, the above four, which have been further optimized, plus scrotal swelling and neonatal conjunctivitis (13). These guidelines, sometimes slightly adjusted to local settings, are still used by many less-resourced countries for the management of STIs (1, 11, 14–16). Briefly, the syndromic management consists of grouping similar symptoms and signs caused by different STI pathogens and then using a combination of antimicrobial agents to treat these STIs (11, 13, 15, 17). Syndromic management is inexpensive, easy to perform and can be useful for immediate treatment of symptomatic patients at the point of care (POC). However, the diagnostic accuracy of the syndromic management of vaginal discharge has been shown highly suboptimal and a majority of urogenital chlamydial, gonorrhoea, and trichomoniasis cases are not detected and treated (because they are asymptomatic), while many cases are unnecessarily or incorrectly treated due to a suboptimal specificity (11, 14, 18–27), and *Mycoplasma genitalium* infections are not taken into account. The reasons for the poor performance of the syndromic management of vaginal discharge include both that STIs are frequently asymptomatic in women (1, 11, 17, 19, 23, 24, 28–30), and that vaginal discharge can additionally be caused by, for example, bacterial vaginosis and candidiasis (1, 11).

Accordingly, the syndromic management of vaginal discharge results in lack of treatment, incorrect treatment, and/or over-treatment, which promote the emergence of antimicrobial resistance (AMR) in STI agents, other pathogens and bystander commensal bacterial species, as well as negatively affect the normal vaginal microbiome (11, 14, 16, 26, 31). The increasing rates of AMR in *N. gonorrhoeae* and *M. genitalium* have made therapeutic options limited, and treatments of these and other STIs should ideally be informed by etiologic diagnoses (1, 17, 20, 32–34).

In Mozambique, as in most other Sub-Saharan African countries, only syndromic management of STIs has been used for more than three decades (3, 4, 35, 36). Consequently, data on prevalence of specific STIs, risk factors associated with these STIs, and the etiology of STI syndromes are completely lacking. This information is urgently needed to assess the effectiveness of the syndromic management of STIs, understand the burden of these STIs particularly in women of reproductive age, and inform revisions of STI treatment guidelines (37).

**Figure 1:**
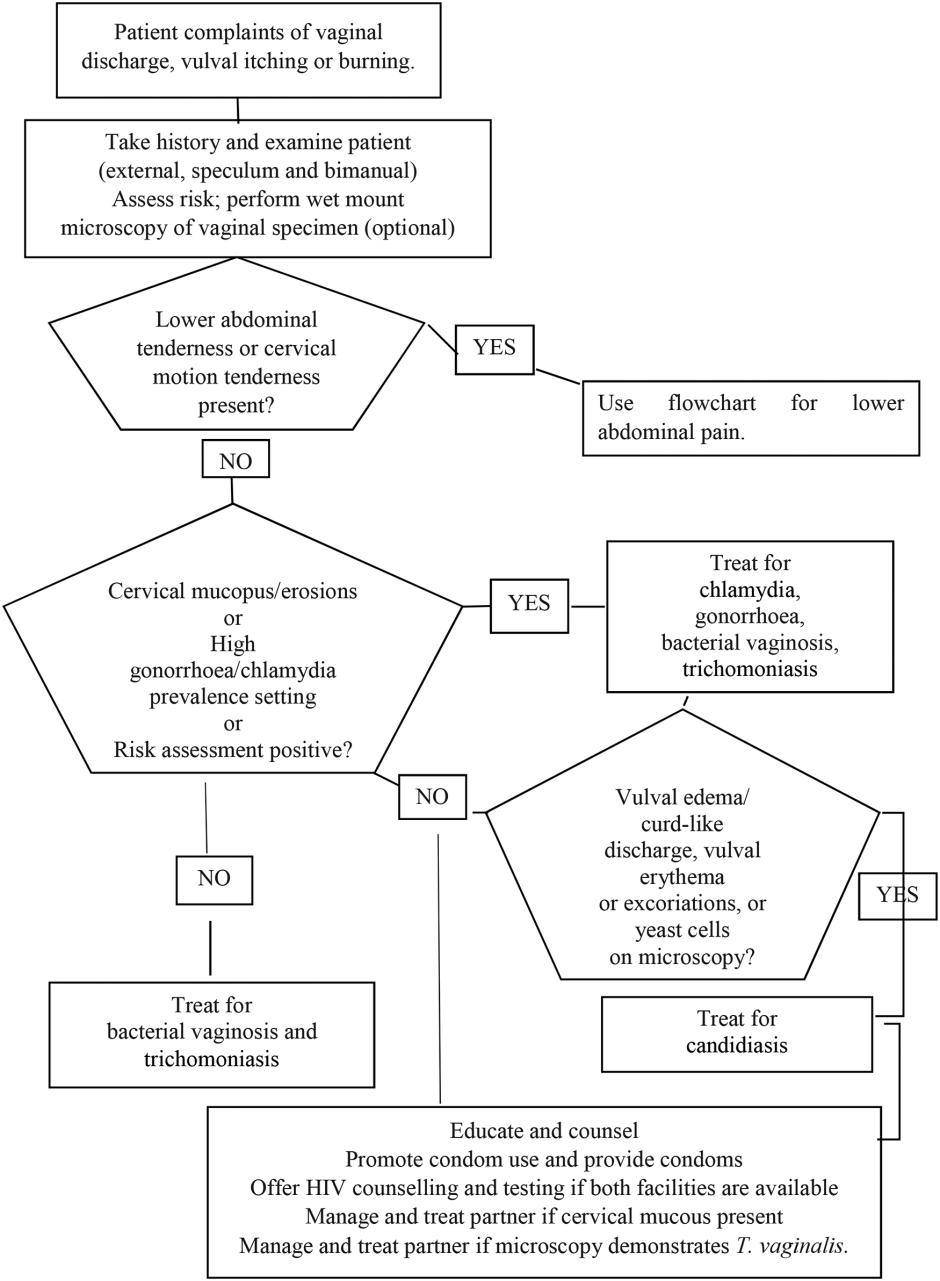
Flowchart for the management of vaginal discharge with bimanual and speculum examination.

The aims of the present study were to examine women in reproductive age with urogenital complaints in Maputo, Mozambique to: (i) describe the prevalence of trichomoniasis, chlamydia, gonorrhoea, and *M. genitalium* infections using a real-time multiplex PCR (38) and HIV using serology, (ii) identify the sociodemographic and behavioural factors among the women associated with these STIs, and (iii) assess the accuracy (sensitivity and specificity) of the vaginal discharge syndrome management in use in Mozambique (Figure 1) to diagnose STIs and reproductive tract infections.

## Material and methods

### Study design, site, and population

This cross-sectional study was carried out in primary healthcare units belonging to the Mavalane sanitary area in Maputo, Mozambique, from February 2018 to January 2019, when 924 women were recruited. The Mavalane sanitary area comprises 12 health centres, namely Polana Caniço, Polana Cimento, 1° de Maio, 1° de Junho, Mavalane, Albazine, Romão, Pescadores, Hulene, Malhagalene, Inhaca, and Katembe. The province of Maputo had a population of about 2.22 million in 2017, of which 1.07 million were men and 1.15 million were women (39), with an estimated HIV-1/2 prevalence of 15.0% in women of reproductive age in 2021 (40).

Since maternal health nurses are generally the frontline health care providers for women with urogenital complaints, cervical cancer screening, and prenatal and family planning consultations in Mozambique, they were eligible researchers. Consequently, the selection of study sites was based on the existence of conditions for gynaecological examination and the availability of maternal health nurses as the main providers of reproductive health services for women. Thus, one hospital [Mavalane General Hospital (suburban)] and three primary health centres, i.e., 1° Junho Health Centre (rural), 1° Maio Health Centre (urban) and, Polana Caniço Health Centre (suburban) were selected because they were having the highest number of consultations for patients with gynaecological complaints.

A patient was eligible if she was between 18 and 49 years of age, had any urogenital complaints such as vaginal discharge, vaginal itching with/without burning, dyspareunia (pain during sexual intercourse), and/or lower abdominal pain, and signed the informed consent form. Fingerprints were collected for those who did not know how to sign. Women who were pregnant, menstruating at the time of consultation, had a positive cervical cancer screening result, reported having used antibiotics or vaginal douches in the last 15 days, had an indication for gynaecological surgery or had symptoms indicating the development of PID, such as spontaneous pelvic pain, were excluded from the study. All participants received counselling before and after a gynaecological examination by community health workers at each study site.

### Data collection and procedures

Investigators were trained in study procedures, data collection, and updated on WHO syndromic management for vaginal discharge.

Sociodemographic, behaviour and clinical data, such as previous STI infections, genital hygiene habits, STI symptoms at the time of interview, number of sexual partners in the last three months, age at first sexual intercourse, and others, were collected at enrolment using a framework standardized questionnaire.

Physical and gynaecological examinations, including speculum examination, were performed. All clinical information was recorded and transferred to the questionnaire. During the speculum examination, samples were collected from the lateral and posterior vaginal fornix and the endocervical canal according to standard procedures. Cell phone numbers were also recorded to allow for follow-up care. Women with symptoms of vaginal discharge syndrome were treated free of charge during the visit according to the national syndromic management guidelines (Figure 1), and women without symptoms of vaginal discharge syndrome were treated based on the results of Gram-stained microscopy.

### Ethical approval

Ethical approval for the study was obtained from the Mozambican National Bioethics Committee for Health (reference number 405/CNBS/2014). This approval has been renewed annually with the current renewal number ref 180/CNBS/23.

### Biological specimens

For the diagnosis of the non-viral STIs, from each participant, specimens from the lateral posterior vaginal fornix and endocervix were collected using sterile swabs, placed in Amies liquid transport medium, and frozen at −70°C. The vaginal/endocervical swab specimens were subsequently sent frozen on dry ice to the WHO Collaborating Centre for Gonorrhoea and Other STIs, Örebro University Hospital, Sweden for DNA extraction and multiplex PCR for diagnosis of chlamydia, gonorrhoea, trichomoniasis and *M. genitalium* infection. For diagnosis of HIV-1/2, 10 ml of venous blood was collected, using the vacutainer system. All samples were placed in a thermal box with ice blocks and transported daily to the Microbiology Laboratory of the Faculty of Medicine of the Eduardo Mondlane University, Maputo, Mozambique, where they were stored at −70°C prior to processing.

### Serological diagnosis of HIV-1/2

Whole blood samples were centrifuged at 8,000 rpm for 10–15 min. HIV-1/2 diagnosis was performed using the national serological testing algorithm, which is based on the use of two 3rd-generation rapid diagnostic tests (RDTs), namely, Alere Determine™ HIV-1/2 (Alere Medical Co. Ltd, Japan) and Uni-Gold™ HIV-1/2 (Trinity Biotech, Wicklow, Ireland). Discordant results were retested using the Enzygnost® HIV-1/2 Integral 4 ELISA (Siemens Healthcare Diagnostics Products GmbH, Marburg, Germany).

### DNA extraction

DNA was extracted from all the vaginal/cervical swab specimens using the QIAamp® DNA Mini Kit (Qiagen, GmbH, Hilden, Germany), in accordance with the manufacturer's instructions. Briefly, 300 µl of each vaginal/cervical swab specimen was centrifuged for 5 min at 5,000 × g (7,500 rpm), and the pellets were used for DNA extraction and purification with the QIAamp® DNA Mini Kit (Qiagen). The final elution was performed using 50 µl of elution buffer. The DNA extracts were stored at −20°C prior to real-time PCR (see below).

### Multiplex diagnostic PCR assay for diagnosis of gonorrhoea, chlamydia, trichomoniasis, and *M. genitalium* infections

The CE/IVD-marked multiplex real-time AmpliSens *N.gonorrhoeae*/*C.trachomatis*/*M.genitalium*/*T.vaginalis*-MULTIPRIME-FRT PCR assay (InterLabService Ltd, Moscow, Russia) was performed, according to the manufacturer's instructions, for diagnosis of gonorrhoea, chlamydia, trichomoniasis and *M. genitalium* infections. This multiplex PCR assay has been previously evaluated with adequate results compared to the Aptima Combo 2 assay (detecting *C. trachomatis* and *N. gonorrhoeae*), Aptima *T. vaginalis* assay and Aptima *M. genitalium* assay (Hologic, San Diego, California, USA) (38). The AmpliSens *N.gonorrhoeae/C.trachomatis/M.genitalium/T.vaginalis*-MULTIPRIME-FRT PCR assay (InterLabService) is based on the amplification of regions of *N. gonorrhoeae*, *C. trachomatis*, *M. genitalium*, and/or *T. vaginalis* DNA using specific primers, which have been previously described (38). The PCR amplified product(s) is detected using fluorescent dyes linked to specific oligonucleotide probes, which bind specifically to the amplified product during PCR thermocycling, and these probes have also been previously described (38). Additionally, an internal control is added to the extraction procedure to control the extraction process and identify possible PCR inhibition. Briefly, 10 µl of extracted DNA template was added to each reaction tube and the final reaction volume was 25 µl. All PCR amplification reactions were carried out on the Rotor-Gene Q 6000 (QIAGEN, Hilden, Germany).

### Syndromic diagnosis

By the syndromic approach, women who had vaginal discharge, itching, or burning in the vulva, without lower abdominal pain were classified as having vaginal and/or cervical infection and received treatment for gonorrhoea, chlamydia, trichomoniasis, and bacterial vaginosis (Figure 1).

### Clinical examination

An external physical examination of the vulva and examination of the endocervix using a speculum was performed.

### Statistical analysis

Data analysis was performed using multiple logistic regression in STATA/IC 12.1 (StataCorp LP, USA) with calculation of a 95% confidence intervals and odds ratio (OR). Pearson's *χ*^2^ test or Fisher's exact test (<5 observations) was used to determine statistically significant relationships between categorical variables. Significance was set at a *p*-value <0.05 for inference.

## Results

### Sociodemographic profile

From February 2018 to January 2019, 1,048 women met the inclusion criteria. Of these, 47 (4.5%) women were excluded because they were menstruating at the time of consultation (*n* = 13), had used antimicrobials in the last 15 days (*n* = 11), were pregnant (*n* = 8), did not consent (*n* = 6), or accepting the gynaecological examination using speculum (*n* = 9). Seventy-seven additional cases were excluded from data analysis because they were missing sociodemographic and behavioural data or appropriate samples. The sociodemographic and behavioural characteristics of the final study participants (*n* = 924) are summarized in Table 1. Briefly, the age of the participants ranged from 18 to 49 years, with a median (mean) age of 28 (30) years. Many participants were under 24 years old (38.9%), more than half were single (50.8%), had secondary school level of education (54.4%), lived in rural areas (54.0%), and had their sexual debut between 12 and 17 years of age (62.1%) (Table 1).

**Table 1:** Sociodemographic characteristics and prevalence of HIV-1/2 in sexually active women (*n* = 924) with urogenital complaints in Maputo, Mozambique.

Variables	Number (%)	HIV-1/2 infection positive rate (%)	*p*-value
Age (years)	* *	* *	**<0** **.** **001**
≤24	359 (38.9)	31 (8.6)	
25–34	282 (30.5)	72 (25.5)	
≥35	283 (30.6)	103 (36.4)	
Place of residence	* *		**<0** **.** **001** *
Urban	53 (5.7)	2 (3.8)	
Rural	499 (54.0)	108 (21.6)	
Suburban	326 (35.3)	52 (16.0)	
Outside of Maputo province	46 (5.0)	44 (95.7)	
Marital status	* *		**<0** **.** **001**
Single	469 (50.8)	87 (18.6)	
Married/Cohabiting	408 (44.2)	95 (23.3)	
Divorced/Separated/Widow	47 (5.1)	24 (51.1)	
Education	* *		**<0** **.** **001**
Primary school/Illiterate	140 (15.2)	62 (44.3)	
Secondary school	503 (54.4)	110 (21.9)	
Higher education	281 (30.4)	34 (12.1)	
Age (years) at first intercourse	* *		**0.003** *
Do not remember	12 (1.3)	4 (33.3%)	
12–17	574 (62.1)	148 (25.8)	
18–23	330 (35.7)	53 (16.1)	
≥24	8 (0.9)	1 (12.5)	
No. of sexual partner in the last three months			0.306
1	870 (94.2)	197 (22.6)	
2 or more	54 (5.8)	9 (16.7)	
Condom use at last sexual intercourse	* *		**0.021**
No	588 (63.6)	117 (19.9)	
Yes	336 (36.4)	89 (26.5)	
Use of contraception method	* *		**0.001**
No	152 (16.5)	50 (32.9)	
Yes	772 (83.2)	156 (20.2)	
Type of contraception used	* *		**0.018**
Did not use any	152 (16.5)	46 (30.3)	
Pill	237 (25.6)	37 (15.6)	
Injection	105 (11.4)	28 (26.7)	
Implant	325 (35.2)	75 (23.1)	
Intrauterine device	75 (8.1)	14 (18.7)	
Others	30 (3.2)	6 (20.0)	
Frequency of vaginal douching	* *		0.071*
Do not practise	81 (8.8)	26 (32.1)	
Daily	828 (89.6)	176 (21.3)	
Sometimes douche	15 (1.6)	4 (26.7)	
Total	**924.0**	**206/924 (22.3)**	

Bold letters indicate statistical significance.

* 2-sided *p*-value of a Fisher's exact test.

### Risk behavioural and clinical findings, and association with HIV-1/2 infection

A high proportion of participants presented with vaginal discharge syndromes (85.0%), followed by lower abdominal pain complaints (11.3%). Other common clinical conditions were cervical ectopia (17.4%) and a low percentage had genital warts (1.2%). The prevalence of HIV-1/2 infection was 22.3% (95% CI: 20%–25%).

Several sociodemographic and/or behavioural variables of the study participants showed a significant association with HIV-1/2 infection, Table 1. Regarding HIV-1/2 infection, the proportions of positivity were significantly higher among women aged ≥35 years than among younger groups (36.4% (103/283) vs. 8.6% (31/359) and 25.5% (72/282), respectively, *p* < 0.001); among those who lived outside Maputo province than among Maputo residents (95.7% (44/46) vs. urban 3.8% (2/53), rural 21.6% (108/499), and suburban 16.0% (52/326), respectively, *p* < 0.001); among the divorced/separated/widowed than among the single and married/cohabiting (51.1% (24/47) vs. 18.6% (87/469) and 23.3% (95/408), respectively, *p* < 0.001); among those with only primary school or being illiterate than those with secondary school or higher education (44.3% (62/140) vs. 21.9% (110/503) and 12.1% (34/281), respectively, *p* < 0.001); among women having their first intercourse at the age of 12–17 years compared to 18–23 years and ≥24 years (25.8% (148/574) vs. 16.1% (53/330) and 12.5% (1/8), respectively, *p* = 0.003); among those using condom at their last sexual intercourse (26.5% (89/336) vs. 19.9% (117/588), *p* = 0.021); and among women not using contraception (32.9% (50/152) vs. 20.2% (156/772), *p* = 0.001). Notably, after excluding the 46 women from outside of Maputo province (95.7% HIV positivity, which was likely because many were referred to Maputo for higher level of health care), most of the significant associations remained. However, HIV positivity was no longer associated with lower age (12–17 years) at the first intercourse (*p* = 0.15) or with not using contraception (*p* = 0.16). Furthermore, the HIV prevalence decreased from 22.3% (206/924) to 18.5% (162/878).

### Prevalence of chlamydia, trichomoniasis, gonorrhoea and *Mycoplasma genitalium* infections stratified by sociodemographic characteristics and sexual behaviour

The prevalence of chlamydia, trichomoniasis, gonorrhoea and *M. genitalium* infections was 15.5% (95% CI: 13%–18%); 12.1% (95% CI: 10%–14%), 4.0% (95% CI: 3.0%–5.0%), and 2.1% (95% CI 1.0%–3.0%), respectively (Table 2). The proportion of concomitant non-viral STIs was as follows: chlamydia/gonorrhoea 1.5%, chlamydia/trichomoniasis 1.5%, chlamydia/*M. genitalium* 0.8%, gonorrhoea/trichomoniasis 0.9%, and trichomoniasis/*M. genitalium* 0.4%.

**Table 2:** Prevalence of STIs and bivariate tests of association between the etiological diagnosis of the four STIs, and sociodemographic, behavioural and clinical variables, among sexually active women with urogenital complaints in Maputo, Mozambique.

	*C. trachomatis*		*T. vaginalis*		*N. gonorrhoeae*		*M. genitalium*	
	Pos (%)	*p*-value	Pos (%)	*p*-value	Pos (%)	*p*-value	Pos (%)	*p*-value
Age group of study participants		0.4		0.7		0.4		0.9
≤24 years	17.3		11.1		5.0		1.9	
25–34 years	15.2		13.1		3.2		2.1	
≥35 years	13.4		12.4		3.5		2.1	
Place of residence		0.8		0.7*		0.6*		0.3*
Urban	13.2		15.1		1.9		3.8	
Rural	16.4		11.4		4.4		1.6	
Suburban	14.1		13.2		4.3		2.8	
Outside of Maputo province	17.4		8.7		0.0		0.0	
Marital status		0.1		0.5*		0.6*		0.4*
Single	17.9		11.9		3.6		1.7	
Married/Cohabit	13.0		13.0		4.7		2.7	
Divorced/Separated/Widow	12.8		6.4		2.1		2.1	
Level of education		0.2		0.1		0.5		0.5*
Primary school	20.0		10.7		2.9		2.9	
Secondary school	14.1		10.3		3.8		1.6	
Higher school	15.7		16.0		5.0		2.5	
Age group at first intercourse		**0.02** *		0.1*		1.0*		0.8*
12–17 years	18.1		10.5		4.2		2.3	
18–23 years	11.2		15.2		3.9		1.8	
≥24 years	0.0		0.0		0.0		0.0	
Do not remember	16.7		16.7		0.0		0.0	
Number of sexual partners in the last three months		**0.01** *		0.5		0.3*		1.0*
1	16.2		12.0		3.8		2.1	
2 or more	3.7		14.8		7.4		2.1	
Self-report use of condom in last sexual intercourse		0.2		0.4		0.9		0.3
No	16.7		12.8		4.1		1.7	
Yes	13.4		11.0		3.9		2.7	
Use of contraceptive methods		0.07		0.1		0.5*		1.0*
No	20.4		8.6		2.6		2.0	
Yes	14.5		12.8		4.3		2.1	
Type of contraceptive method		0.6*		0.1		0.7*		0.5*
Did not use any	19.7		9.2		2.6		2.0	
Pill	13.5		15.6		3.4		3.4	
Injection	15.2		14.0		2.9		0.0	
Implant	15.1		11.1		5.5		2.2	
Intrauterine device	17.3		6.7		4.0		1.3	
Other methods	10.0		20.0		3.3		0.0	
Vaginal discharge syndromes		0.4		0.2		**0.04**		0.2*
No	18.0		15.1		7.2		3.6	
Yes	15.0		11.6		3.4		1.8	
Frequency of vaginal douching		**<0.001** *		0.8		0.4*		0.8*
Do not practise	32.1		12.3		4.9		2.5	
Daily	14.2		12.2		3.9		2.1	
Sometimes douche	0.0		6.7		6.7		0.0	
Low abdominal pain syndrome		0.6		0.2		1.0*		1.0*
No	15.2		11.6		4.0		2.1	
Yes	17.3		16.3		3.8		1.9	
Dyspareunia		0.5		0.9		**<0.001**	** **	**0.03** *
No	15.3		12.1		3.5		1.8	
Yes	20.0		11.4		17.1		8.6	
Cervical inflammation present		0.2		0.5		0.6		1.0
No	17.0		12.8		4.4		2.1	
Yes	13.5		11.3		3.5		2.0	
Cervical ectopia observed		0.8		0.9		0.3		1.0*
No	15.3		12.2		3.7		2.1	
Yes	16.1		11.8		5.6		1.9	
Vulva inflammation		0.7		0.7		0.7		0.8*
No	15.3		11.3		3.9		2.2	
Yes	15.8		16.6		4.6		1.3	
Genital warts		0.7*		1.0*		1.0*		1.0*
No	15.4		12.2		4.1		2.1	
Yes	18.2		9.1		0.0		0.0	
HIV-1/2 infection		0.9		1.0		0.9		0.3*
No	15.6		12.1		4.0		2.4	
Yes	15.0		12.1		3.9		1.0	

Positive cases.

Bold letters indicate significance.

* 2-sided *p*-value of a Fisher's exact test.

The proportions of *C. trachomatis* cases were significantly higher among women with early sexual debut (12–17 years, 18.1% (104/574) vs. 11.2% (37/330) and 0.0% (0/20), respectively, *p* = 0.02), in women who had a single sexual partner in the last three months (16.2% (141/870) vs. 3.7% (2/54), *p* = 0.01), and in women that did not practice vaginal douching daily (32.1% (26/81) vs. 14.2% (117/826), *p* < 0.001), Table 2. The proportions of gonorrhoea cases were significantly higher among women who did not have vaginal discharge syndrome (7.2% (10/139) vs. 3.4% (27/785), *p* = 0.04), and among those who reported dyspareunia (17.1% (6/35) vs. 3.5% (31/889), *p* < 0.001). Regarding *M. genitalium* infection, a higher proportion of positive cases was found only among women who reported dyspareunia (8.6% (3/35) vs. 1.8% (16/889), *p* = 0.03). Trichomoniasis was not significantly associated with any of the examined variables, Table 2. All these associations remained significant after excluding the 46 women from outside of Maputo province (95.7% HIV positivity, which was likely because many were referred to Maputo for higher level of health care).

### Sensitivity and specificity of the vaginal discharge syndrome management compared with laboratory-based etiological diagnoses

Supplementary Table S1 summarizes the accuracy (sensitivity and specificity) of the vaginal discharge syndrome management compared with the etiological laboratory diagnosis of the four STIs.

Briefly, the syndromic approach based on vaginal symptoms or signs (Figure 1) had a sensitivity ranging from 73.0% to 82.5% and specificity of 14%–15% in the detection of women with urogenital chlamydia, trichomoniasis, gonorrhoea, or *M. genitalium* infection. The sensitivity and specificity of the vaginal syndromic approach to detect women with any of the three STIs chlamydia, trichomoniasis and gonorrhoea (which are treated in the vaginal discharge syndromic management) was 80.8% and 13.1%, respectively. Consequently, 19.2% of the women with any of these three STIs would not be detected and accordingly treated using the vaginal discharge syndromic management (missed treatment) and 70.0% of the women would be treated despite not infected with any of these three STIs (overtreatment). The sensitivity and specificity for detection of the different single STIs ranged from 73.0% to 82.5%, and from 14.5% to 14.8%, respectively (Supplementary Table S1).

## Discussion

We investigated the prevalence of STIs (chlamydia, gonorrhoea, trichomoniasis, *M. genitalium* and HIV-1/2) in women with urogenital complaints that attended for care at four different health facilities in Maputo province, Mozambique. A high prevalence of non-viral STIs, and HIV-1/2 infection was observed, with chlamydia being the most common non-viral STI, followed by trichomoniasis. Risk factors associated with STIs included younger age at first sexual intercourse, not using vaginal douching, absence of vaginal syndrome and dyspareunia. Older age of the women, living in non-urban ares, being divorced/separated/widowed, having low level of education, and condom use at last sexual intercourse were associated with HIV-1/2 positivity. Furthermore, we show that the syndromic management of vaginal discharge has a low accuracy in the detection of STIs in Mozambique, especially low specificity, which results in under-treatment of STI-positive cases and incorrect or over-treatment of women with urogenital complaints, many of whom are negative for all the non-viral STIs. Finally, the resistance to ciprofloxacin in *N. gonorrhoeae* is exceedingly high globally (33) and it is imperative that ciprofloxacin is replaced by, for example, ceftriaxone 500 mg in the syndromic management of vaginal discharge as well as in other syndromic management flowcharts used in Mozambique.

Comparing to some previous studies on symptomatic women in other sub-Saharan African countries, such as Zimbabwe (41), South Africa (42) as well as Kenya (31), the prevalence of chlamydia (15.5%) and trichomoniasis (12.1%) in the present study were similar. However, these prevalences were lower than those previously found in pregnant women in South Africa (43) and higher than that recorded in Senegal in symptomatic women (44). The prevalence of gonorrhoea, however, was consistent with that reported among pregnant women in Ethiopia (45), but lower than that among symptomatic women in Kenya (46), women of reproductive age in Swaziland (47), and young women in Zimbabwe and South Africa (48). However, the gonorrhoea prevalence was higher than those recorded, for example, among female students in Tanzania (49). High prevalences of the most common non-viral STIs (chlamydia, gonorrhoea, trichomoniasis and *M. genitalium* infection) were also found in studies including a mixed population in Botswana, Namibia, and South Africa (6). *M. genitalium* infection was less common in our study, which was inconsistent with results reported in Kenya among women with lower genital tract symptoms (46).

The proportions of non-viral STI co-infections were comparable to those reported in previous community-based randomised trials and cross-sectional studies, carried out in Guinea-Bissau (50), Ethiopia (45), Kenya (Maina et al., 2016), Australia (51) and Brazil (52), in which the prevalence of concomitant STIs infections in women were 0.6%–2.0% of chlamydia/gonorrhoea, 0.3%–2.8% of chlamydia/trichomoniasis, 0.2%–1.3% of chlamydia/*M. genitalium* and 0.2%–2.4% of gonorrhoea/trichomoniasis. These results suggest that the proportions of non-viral STI co-infections may vary but are in general low in this population. Results are also influenced by the diagnostic methods used and which population groups that have been studied. It is frequently higher in specific risk groups such as sex workers (53) and women living with HIV-1/2 (43, 54).

For non-viral STIs, we identified an association between chlamydial infection and sexual behaviour, with early age of sexual debut and/or number of sexual partners being risk factors for this infection. These findings were in agreement with previous studies (42, 55), which reported an increased risk of infection in women who initiated sexual activity under the age of 18 years. Discussing relations is an extremely private, sensitive issue and considered a taboo subject in very conservative contexts in Mozambique. Thus, the high prevalence of chlamydia infections in participants who reported having had only one sexual partner in the last three months may be attributed to a social desirability bias (42, 56, 57), however, we cannot exclude that some of these women were aware of their risk behaviour and more regularly used condoms to protect themselves during sexual intercourse. There were no significant differences in STI burden between women living with HIV-1/2 compared to other women, i.e., in contrast to the findings of previous studies in which STI prevalence was higher among African women living with HIV-1/2 (43, 54, 58–64). This may reveal different risk behaviours among populations in different countries that increase the risk of contracting HIV-1/2 and STIs. Previous studies (65–68) have stated that individuals over 20 years of age were more likely to test positive for HIV-1/2 than adolescents (15–19 years), which is consistent with our findings. In general, more adults engage in risky sexual behaviour, for many reasons, than adolescents, in addition to most likely having more sexual experiences in their longer life and a higher risk of being infected. Since sexual intercourse is the main route of HIV-1/2 transmission, the risk in adults is even greater (66). Furthermore, being divorced/separated/widowed, uneducated, or less educated was associated with higher HIV-1/2 prevalence, which is consistent with previous studies in Mozambique (66), and in other countries (69, 70). People with lower education are less likely to seek information about healthcare and therefore more likely to have poor health outcomes (71).

However, several sociodemographic factors, sexual behaviour and contraceptive use did not show any associations with the presence of single or concomitant non-viral STIs. This has been shown also in other studies in sub-Saharan Africa in different population groups, which concluded that age (31, 42, 72), place of residence (72), level of education (73), marital status (31), contraceptive use and lifetime partners (73) were not risk factors for a single or co-infections with non-viral STIs. Nevertheless, in populations living with HIV in the African region, multiple sexual partners (74–76), lack of condom use and low level of education (49, 77) have been highlighted as risk factors for viral as well as non-viral STIs. Correct condom use is known to be very effective in preventing the transmission of HIV-1/2 and other STIs (78, 79). We found a lower prevalence of women living with HIV-1/2 among women who reported not using a condom at last intercourse, which has also been described among women in South Africa (80). These results indicate that condom use during intercourse may not have been a consistent practice in their sexual relationships, and perhaps condom use, regular and irregular, indicates a higher sexual risk behaviour (more partners, more casual sex, sex work etc.) resulting in higher HIV-1/2 prevalence in this group. In addition, it has been reported that most women in sub-Saharan Africa are unable to negotiate with their sexual partners for the consistent use of male or female condoms (81). Understanding the cultural and social factors that determine sexual partnerships in Mozambique may be useful in identifying why women are more likely to be infected with HIV-1/2 and STIs.

Although vaginal discharge syndrome was the most common complaint of the participating symptomatic women, we found no significant positive association with any of the four non-viral STIs, which is in line with previous studies in the sub-Saharan region (42, 46), but inconsistent with previous findings from Ethiopia (45). Vaginal douching or cleansing with soap, water or other substances is a common practice among women in many Sub-Saharan African countries (82–84), including Mozambique. In the present study, there was no positive association between vaginal douching and any STIs/HIV. However, several other studies have shown that frequent vaginal douching can be a risk factor associated with STIs, including HIV-1/2 (83, 85–88). This may be because it causes changes in the normal vaginal flora or causes dryness or burning, disturbance and inflammation of the mucous membranes (89). The vaginal douching practice has also been shown to promote the spread of microorganisms to the upper genital tract (84, 90, 91).

In our study, the sensitivity of the vaginal discharge syndrome algorithm to detect the four diagnosed non-viral STIs was relatively high (reflecting the studied population of women with urogenital complaints), but the specificity was very low, resulting in high rates of overtreatment and incorrect treatment, as most women reporting vaginal syndromes did not require treatment because they did not have an STI and instead may have been suffering from bacterial vaginosis, Candida vaginitis, or similar. The inclusion of risk assessment in the syndromic management of vaginal discharge has been shown to decrease overtreatment in pregnant and non-pregnant women in Tanzania (92). This is because women with vaginal discharge would only receive treatment for chlamydia and gonococcal infection if the risk assessment is positive. In Mozambique, the diagnosis of non-viral STIs in women is based entirely on self-reported vaginal discharge syndromes and the women's physical examination when necessary. However, in most health facilities providing primary health care in Mozambique, physical examination with a genital speculum is not routinely performed due to lack of examination rooms and adequate equipment such as gynaecological beds, speculum, autoclaves, etc., in addition to limited and overstretched human resources.

Also in previous studies, the vaginal discharge syndrome approach has demonstrated low diagnostic accuracy, i.e., with sensitivity ranging between 14 and 92.9%, specificity varying between 2.4%–33% and PPV of 8%–38% (31, 72, 93) in diagnosing non-viral STIs in women with urogenital complaints. Indeed, several authors have reported the poor performance of vaginal discharge syndrome approaches in the diagnosis of non-viral STIs in different regions (11, 21, 22, 25, 27, 36, 94–97). The lack of detection of asymptomatic STIs (11, 22, 75, 98, 99), risk of selecting AMR due to the overtreatment, which is linked to the low specificity, strongly emphasize the need for etiological STI diagnosis in women with urogenital complaints as well as asymptomatic women that have been exposed to a risk of being infected by an STI.

Rapid point-of-care tests (POCTs) for HIV-1/2 and syphilis have already been successfully integrated into many antenatal screening programmes in several Sub-Saharan African countries aiming to eliminate mother-to-child transmission of HIV-1/2 and syphilis (100–105). While there are available adequate POCTs for the two non-viral STIs syphilis and trichomoniasis, there are currently no appropriate rapid, affordable, and effective POCTs for chlamydia, gonorrhoea, or *M. genitalium* infection (106, 107). Accordingly, rapid, affordable, and effective POCTs for all non-viral STIs are essential to develop for detection of these infections in symptomatic and asymptomatic males and females. Several such POCTs are in the pipeline, especially for chlamydia and gonorrhoea (106).

## Strengths and limitations

This was the first study to evaluate the accuracy of any of the syndromic management flowcharts for STIs in Mozambique, and previously evaluated, standardized and quality-assured techniques and assays for etiological diagnosis were used. However, the study also included some limitations. First, we used a cross-sectional study design and non-random convenience sampling, which may have introduced some biases in the measurement of certain variables. Second, the selection of participants with urogenital complaints and the exclusion of 75 women due to missing sociodemographic and behavioural data or appropriate samples may have introduced selection biases. Accordingly, a large study using population-based sampling and including both symptomatic and asymptomatic women with less women excluded would be ideal to provide more accurate STI prevalence figures and sensitivity and specificity of the vaginal discharge syndrome flowchart used in Mozambique. This study should also ideally include antimicrobial susceptibility testing for *N. gonorrhoeae* and *M. genitalium*, which would provide data to inform recommended treatment and such data are totally lacking in Mozambique. Furthermore, it is important to evaluate, compared to etiological diagnosis, the accuracy of also the other STI-related syndromic management flowcharts in Mozambique, i.e., urethral discharge, genital ulcer, lower abdominal pain or pelvic inflammatory disease, and neonatal conjunctivitis flowcharts. Finally, in the present study the data regarding sociodemographics, sexual behaviour and symptoms were self-reported and may potentially be prone to social desirability bias and recall bias.

## Conclusions

In conclusion, we found a high prevalence of especially chlamydia, trichomoniasis, and HIV-1/2 in women with urogenital complaints in reproductive age, mostly single, with secondary schooling, and with sexual debut between 12 and 17 years old in Maputo, Mozambique. Syndromic management of vaginal discharge revealed low accuracy for diagnosis of non-viral STIs, especially low specificity, resulting in undertreatment of STI-positive cases and incorrect or overtreatment in STI-negative cases, since many women with vaginal discharge syndromes were negative for all tested non-viral STIs and likely suffered from other etiologies, for example, bacterial vaginosis. Our findings strongly emphasize the urgent need for enhanced etiological testing of non-viral STIs in less-resourced settings and, ideally, rapid, affordable, and accurate POCTs for diagnosis of non-viral STIs in both symptomatic and asymptomatic individuals. This etiological diagnosis needs to be linked to access to evidence-based appropriate treatment for all those infected, which would require the availability and accessibility of health services in communities that have free effective antimicrobials available. Furthermore, it is important to strengthen prevention strategies to reduce the prevalence of STIs/HIV in this setting. Prevention strategies should include improved sexual health education campaigns (and sexual health education in school), including STIs/HIV, and promoting condom use, with the participation of community health workers, health authorities and researchers. It is also imperative to reduce the stigma and discrimination related to STIs/HIV among women and young adults, as well as to ensure confidentiality and privacy to increase the uptake of sexual partner notification. Finally, introduction of regular and quality-assured antimicrobial susceptibility surveillance for especially *N. gonorrhoeae*, but ideally also *M. genitalium*, is important to provide evidence-based resistance data that can inform refinements of the treatment recommendations.

## Data Availability

The raw data supporting the conclusions of this article will be made available by the authors, without undue reservation.
