# Supplementary material for: Prevalence of sexually transmitted infections (STIs), associations with sociodemographic and behavioural factors, and assessment of the syndromic management of vaginal discharge in women with urogenital complaints in Mozambique
Source: Front Reprod Health. 2024 Apr 18;6:1323926. doi: 10.3389/frph.2024.1323926 (PMC11067503; doi:10.3389/frph.2024.1323926)
Supplement: Supplementary file 1 [file Table1.docx]

**SUPPLEMENTARY TABLE 1 |** Vaginal discharge syndrome compared to laboratory-based etiological diagnosis of four non-viral STIs among sexually active women with urogenital complaints in Maputo, Mozambique.

|  | **Laboratory diagnosis** | | | | | |
| --- | --- | --- | --- | --- | --- | --- |
| **Clinical condition** | **Pos** | **Neg** | **Sensitivity (%)** | **Specificity (%)** | |  |
| VDS | *C. trachomatis* | | |  |  |  |
| Yes | 118 | 667 | 82.5, 95% CI 75-88% | 14.6, 95% CI 12-17% | |  |
| No | 25 | 114 |  |  |  |  |
| VDS | *T. vaginalis* | | |  |  |  |
| Yes | 91 | 694 | 81.3, 95% CI 73-88% | 14.5, 95% CI 12-17% | |  |
| No | 21 | 118 |  |  |  |  |
| VDS | *N. gonorrhoeae* | | |  |  |  |
| Yes | 27 | 758 | 73.0, 95% CI 56-86% | 14.5, 95% CI 12-17% | |  |
| No | 10 | 129 |  |  |  |  |
| VDS | *M. genitalium* | | |  |  |  |
| Yes | 14 | 771 | 73.7, 95% CI 49-90% | 14.8, 95% CI 13-17% | |  |
| No | 5 | 134 |  |  |  |  |
| VDS | *C. trachomatis/N. gonorrhoeae* | | | |  |  |
| Yes | 145 | 640 | 80.6, 95% CI 74-86% | 14.0, 95% CI 12-17% | |  |
| No | 35 | 104 |  |  |  |  |
| VDS | *C. trachomatis/T. vaginalis/N. gonorrhoeae* | | | | | |
| Yes | 236 | 549 | 80.8, 95% CI 76-85% | 13.1, 95% CI 11-16% | |  |
| No | 56 | 83 |  |  |  |  |
| VDS | *C. trachomatis/T. vaginalis/N. gonorrhoeae/M. genitalium* | | | | | |
| Yes | 250 | 535 | 80.4, 95% CI 75-85% | 12.7, 95% CI 10-16% | |  |
| No | 61 | 78 |  |  |  |  |

*Pos, positive; Neg, negative; VDS, vaginal discharge syndromes (vaginal discharge, itching or burning); CI, confidence interval.*
